# Long-Term Effects of Lifestyle Interventions Targeting Diabetes and Weight Loss on Aging-Related Outcomes

**DOI:** 10.1093/geroni/igaf122.466

**Published:** 2025-12-31

**Authors:** Peter Huckfeldt, Kristine Ensrud

## Abstract

Type 2 diabetes can lead to other chronic conditions (e.g., heart disease) and physical disability, and when paired with obesity, accelerates aging processes and functional decline, threaten independence and quality of life, and increase clinical care needs and complexity. Lifestyle interventions focused on reducing weight and increasing physical activity have been shown to improve diabetes control and functional status and reduce multimorbidity. However, their long-term benefits remain incompletely understood. The Diabetes Prevention Program (DPP) and the Action for Health in Diabetes (Look AHEAD) study examined the effects of lifestyle interventions on delaying the onset of type 2 diabetes and diabetes-related cardiovascular outcomes. Between 1996 and 2021, the DPP randomized 3,234 participants (mean age, 51 yrs) who were at high risk for developing type 2 diabetes to an intensive lifestyle intervention (ILI) arm, metformin, or a control group. Look AHEAD randomized 5,145 participants (mean age, 58.7 yrs) with type 2 diabetes and overweight or obesity to an ILI or a control group between 2001 and 2004. While both interventions concluded over 10 years ago, participants’ health status and health care use has been continuously monitored through a combination of surveys and linkages to Medicare data, providing ideal data for studying the long-term effects of lifestyle intervention. This symposium will present research on the long-term effects of an ILI on multimorbidity in DPP (Salive and colleagues), nursing home residence and institutional days in Look AHEAD (Huckfeldt and colleagues), and mortality in Look AHEAD (Houston and colleagues).

